# Cancer treatment regimens and their impact on the patient-reported outcome measures health-related quality of life and perceived cognitive function

**DOI:** 10.1186/s41687-022-00422-5

**Published:** 2022-02-21

**Authors:** Niklas Paul Grusdat, Alexander Stäuber, Marion Tolkmitt, Jens Schnabel, Birgit Schubotz, Peter Richard Wright, Marc Heydenreich, Dirk-Henrik Zermann, Henry Schulz

**Affiliations:** 1 Professorship of Sports Medicine/Sports Biology, Institute of Human Movement Science and Health, Faculty of Behavioral and Social Sciences, Chemnitz University of Technology, Thüringer Weg 11, 09126 Chemnitz, Saxony/Sachsen Germany; 2 Rotes Kreuz Krankenhaus, Red Cross Hospital, Chemnitz-Rabenstein, Germany; 3 Clinical Cancer Registry, Tumorzentrum Chemnitz e.V., Chemnitz, Germany; 4 Department of Sport, Health Sciences and Social Work, Oxford Brookes University, Oxford, UK; 5 Department of Urology and Urooncology, Vogtland-Klinik, Bad Elster, Germany

**Keywords:** HRQoL, Cognitive function, Survivorship, Support

## Abstract

**Background and purpose:**

Breast cancer can be a significant challenge for those affected. Knowledge of physical function, social-emotional challenges, and perceived cognitive function based on the cancer treatment regimens may help to inform adequate support.

**Methods:**

For this prospective observational pilot study, we collected data of seventy-nine women (mean age 54.6 ± 9.5 years) before (T0) and after (T1) initial breast cancer treatment. Functional Assessment of Cancer Therapy-Breast (FACT-B) and Functional Assessment of Cancer Therapy–Cognitive-Function (FACT-Cog) were used to collect data of four treatment subgroups: SCR = Surgery + Chemotherapy + Radiation Therapy; SC = Surgery + Chemotherapy; SR = Surgery + Radiation Therapy; S = Surgery. A mixed ANOVA and posthoc analysis (Tukey, Games-Howell) were used to detect interactions (group by time) and the main effect. A repeated-measures ANOVA displayed individual group differences (time).

**Results:**

Significant interaction showed more deterioration was experienced with SC and SCR than SR and S for FACT-B (*p* < 0.01) and FACT-Cog (*p* < 0.001). The longitudinal comparison between T0 and T1 indicated a significant group main effect on all subscales (*p* < 0.001) except for Emotional Well-Being. Significant reductions (*p* < 0.05) in FACT-B, (− 19%); FACT-Cog, (− 21%) with most pronounced effect in Physical Well-Being (− 30%), Functional Well-Being (− 20%), Breast Cancer Subscale (− 20%), Perceived Cognitive Impairments (− 18%) and Impact of Cognitive Impairments on Quality of Life (− 39%) were detected for SCR.

**Conclusion:**

Our study showed that the extent of change in health-related quality of life (HRQoL) and perceived cognitive function (PCF) depends on the treatment regimen. Multidisciplinary support initiated early in breast cancer therapy is needed, especially for women undergoing combined cancer treatment. Routine assessment of patient-reported outcomes (PROs) in oncology practice may increase the transparency of patients’ perceived circumstances, leading to personalized and optimized acute and survivorship care.

## Introduction

Female breast cancer is the most commonly diagnosed cancer worldwide, with an estimated 2.3 million new cases in 2020 [1, 2]. About 70,000 new cases are diagnosed in Germany every year [3]. With personalized medicine, overall survival has improved in recent decades, especially for patients with early-stage disease [4].

Scientific research has shown evidence of treatment-associated social-emotional challenges [5, 6], physical-functional limitations [7, 8], and reduced health-related quality of life (HRQoL) [9]. Threats linked to women experiencing breast cancer are chemotherapy-related adverse events, including cognitive impairment such as memory loss, inability to concentrate, difficulty in thinking and processing information [10–12]. Especially at an early stage in life, unmanaged deficits may lead to the inability to function in the workplace or handle instrumental daily living activities, such as finances, shopping, and housekeeping [13, 14].

While studies have examined the impact of breast cancer patients receiving active treatment, there are inconsistencies regarding the application of patient reports in routine oncology practice [15, 16]. Functional Assessment of Cancer Therapy-Breast (FACT-B) and Functional Assessment of Cancer Therapy–Cognitive-Function (FACT-Cog) have gained scientific credibility by providing a comprehensive patient-orientated picture about HRQoL and PCF. Patient-reported outcome (PRO) measures may describe prognostic relevant disease progression, subjective perception of symptoms, the prevalence of satisfaction with care, and the patients’ point of view on health status [17–19]. Close monitoring of the patients’ situation appears relevant before initiating medical treatment and follow-up to support unmet care needs and conduct a risk stratification.

Alongside the traditional clinical reports, the importance of measuring PRO in patients with breast cancer is required to improve the quality of care [20–22].

Differentiating patient-perceived circumstances regarding treatment regimens (chemotherapy, surgery, radiation therapy, endocrine therapy) may personalize and optimize acute and survivorship care. Further carefully observing treatment-specific conditions may guide the decision-making of a multidisciplinary team of health care specialists. The purpose of the present study was to compare self-reported PROs on HRQoL and PCF in women with breast cancer before and after undergoing various breast cancer treatments.

## Methods and ethics

Between April 2018 and April 2020, a total of 120 patients with the first diagnosis of breast cancer were recruited within the research study “Return” (trial acronym), approved by the Ethics Committee. This study involved humans and addressed health issues. Therefore, it was registered with the German Clinical Trials Register (DRKS). The current study was performed in line with the principles of the Declaration of Helsinki (1986).

### Recruitment of patients

All patients were recruited in a Hospital in Chemnitz-Rabenstein, Germany. Within one week after a breast cancer diagnosis, patients were invited for consultation by their oncologist and informed about possible participation in the present study. The Return study is part of a series of projects focusing on supporting the participation in survivorship care measures of offered follow-up rehabilitation therapy. Moreover, ambulant oncological exercise therapy groups in survivorship were set up.

**Fig. 1:**
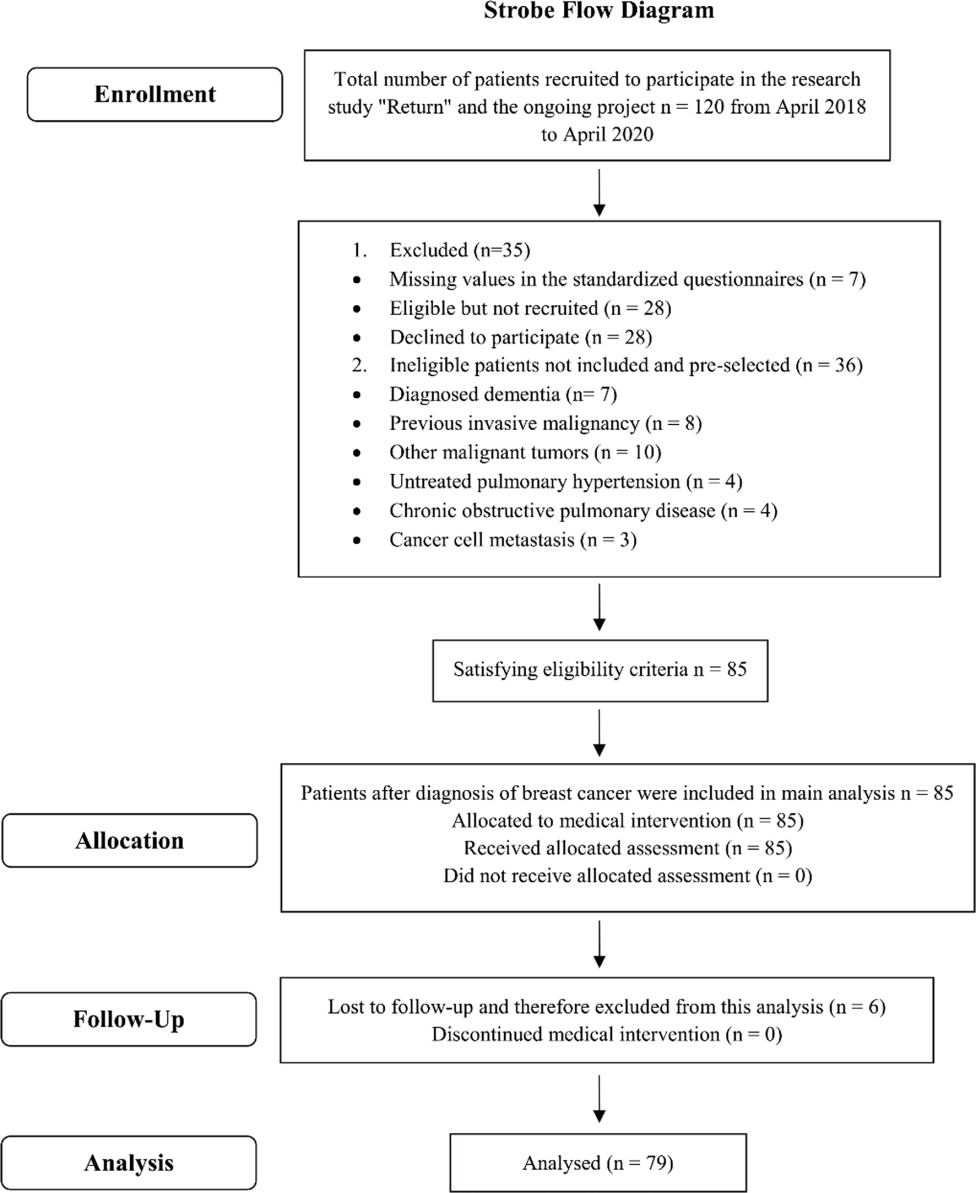
STROBE flow diagram of the prospective observational pilot study in women with breast cancer

Patients were pre-selected and identified as ineligible for participating in this study after checking the medical record and completion of medical history interview with previous invasive malignancy, other malignant tumors, cancer cell metastasis, untreated pulmonary hypertension, diagnosed dementia, and chronic obstructive pulmonary disease. Participants were also excluded due to missing values in the questionnaires, declining consent, or being lost to follow-up. Participants had the opportunity to discuss their participation and read and consider the research information leaflet. A sufficient time (> 24 h) to reflect on the implications of participating in the study was provided. The response rate was 71%. Inclusion criteria for this analysis were—patients’ written informed consent, a recent diagnosis of untreated female breast cancer, age < 70 years. Eighty-five participants who had not initiated cancer treatment met the inclusion criteria and completed the medical interventions. A differentiation into four treatment groups was obtained for statistical analysis of the prospective observational pilot study. Due to ethics and legal considerations, no random assignment occurred. Appointments for the allocated assessments were made immediately and towards the end of medical treatment. Cooperation and coordination between parties involved were required to ensure participation. Further limitations recorded are presented in Fig. 1.

### The return study

According to evidence-based clinical practice guidelines in oncology, every patient could receive the treatment, which was decided by physicians and in line with informed consent and patient agreement. Additionally, this longitudinal study was analyzed by investigators who were not involved in any interventions or clinical decisions to gather information and compare changes over time. Researchers collected data through questionnaires of women with breast cancer to learn more about the effects of different cancer treatments on cognitive health and HRQoL.

### The clinical cancer registry

With the national German cancer registry law, nationwide epidemiological cancer registries were established in 1995. Consequentially, the German National Cancer Plan worked out a legal basis for national clinical cancer registries (CCR). Existing structures were expanded to clinical and epidemiological CRs. A joint evaluation point, a central coordination point, a common Tumor database, and a scientific advisory board were installed.

Physicians and hospitals (service providers) have the statutory duty to notify clinical markers related to cancer, such as tumor localization, tumor pathology, estrogen receptor, progesterone receptor, human epidermal growth factor receptor status, parameters for tumor grading, UICC status, and medical interventions. The optimization of linked datasets offers opportunities for research studies due to access to patient information. All in all, CCRs have the realistic potential to improve oncological health care throughout Germany [3, 23]. Further, joint activities of all health policies and scientific actors involved in the fight against cancer are indispensable to continue withstanding the high pressure to innovate, primarily by targeted oncological drugs.

### Measurements

All assessments were carried out before (T0) and within one week after completing (T1) conventional cancer treatment (surgery, chemotherapy, radiation therapy). Cases with long-term endocrine therapy continued beyond T1. Based on the variable duration of breast cancer treatment for each woman, repeated testing (T0 and T1) was performed at different time intervals. Four treatment subgroups were included for the following analysis (SC, Surgery + Chemotherapy; SCR, Surgery + Chemotherapy + Radiation Therapy; SR, Surgery + Radiation Therapy; S, Surgery). Height and weight were measured with footwear and headwear removed using a standard stadiometer and weigh scale, Seca IEC 601 (Vogel & Halke, Hamburg, Germany). These parameters allowed the calculation of the body mass index (BMI) by using the formula $BMI = \frac{{weight \left( {{\text{kg}}} \right)}}{{height ({\text{m}})^{2} }}$.

### The questionnaires

Eligible patients were asked to fill out the validated German translations of the Functional Assessment of Cancer Therapy-Breast (FACT-B) and the Functional Assessment of Cancer Therapy–Cognitive Function (FACT-Cog) questionnaires. All patients completed the questionnaires with qualified personnel available to answer any questions or clarify any meaning. For FACT-B and FACT-Cog, higher scores (negative items were reverse-scored) indicate a subjectively better HRQoL or PCF. The past seven days as the recall period are covered by both questionnaires, which includes times when patients were undergoing treatment. The FACT-B (version 4) instrument has a score range of 0–148 points and consists of 37 questions and item codes. It is designed to capture five domains in breast cancer patients: Physical Well-Being (PWB 0 to 28), Social/Family Well-Being (SWB 0 to 28), Emotional Well-Being (EWB 0–24), Functional Well-Being (FWB 0–28), and Breast Cancer Subscale (BCS 0–40). For each question of the subscales, a response is required on a 5-point Likert-type scale (0, not at all; 1, a little bit; 2 some-what; 3, quite a bit; 4, very much) (see Table 1) [17, 24].

**Table 1:** Functional Assessment of Cancer Therapy-Breast (FACT-B, version 4)

Subscale	FACT-B (version 4)		
	Item ID	Item/question	Response options
PWB	GP1	I have a lack of energy	Intensity (not at all, a little bit, somewhat quite a bit, very much)
	GP2	I have nausea	
	GP3	Because of my physical condition, I have trouble meeting the needs of my family	
	GP4	I have pain	
	GP5	I am bothered by side effects of treatment	
	GP6	I feel ill	
	GP7	I am forced to spend time in bed	
SWB	GS1	I feel close to my friends	Intensity (not at all, a little bit, somewhat quite a bit, very much)
	GS2	I get emotional support from my family	
	GS3	I get support from my friends	
	GS4	My family has accepted my illness	
	GS5	I am satisfied with family communication about my illness	
	GS6	I feel close to my partner (or the person who is my main support)	
	GS7	I am satisfied with my sex life	
EWB	GE1	I feel sad	Intensity (not at all, a little bit, somewhat quite a bit, very much)
	GE2	I am satisfied with how I am coping with my illness	
	GE3	I am losing hope in the fight against my illness	
	GE4	I feel nervous	
	GE5	I worry about dying	
	GE6	I worry that my condition will get worse	
FWB	GF1	I am able to work (include work at home)	Intensity (not at all, a little bit, somewhat quite a bit, very much)
	GF2	My work (include work at home) is fulfilling	
	GF3	I am able to enjoy life	
	GF4	I have accepted my illness	
	GF5	I am sleeping well	
	GF6	I am enjoying the things I usually do for fun	
	GF7	I am content with the quality of my life right now	
BCS	B1	I have been short of breath	Intensity (not at all, a little bit, somewhat quite a bit, very much)
	B2	I am self-conscious about the way I dress	
	B3	One or both of my arms are swollen or tender	
	B4	I feel sexually attractive	
	B5	I am bothered by hair loss	
	B6	I worry that other members of my family might someday get the same illness I have	
	B7	I worry about the effect of stress on my illness	
	B8	I am bothered by a change in weight	
	B9	I am able to feel like a woman	
	P2	I have certain parts of my body where I experience pain	

The FACT-Cog version 3 consists of 33 questions and item codes to derive a total score (0 to 132). It has four subscales representing PCF and its impact on quality of life: Perceived Cognitive Impairments (PCI 0–72), Comments From Others (OTH 0–16), Perceived Cognitive Abilities (PCA, 0–28), and Impact of Perceived Cognitive Function on Quality of Life (QoL 0–16). The Likert-type scales offer the option to identify patients’ perceived situation regarding frequency and intensity: PCI, OTH = 0, Never; 1, About once a week; 2, Two to three times a week; 3, Nearly every day; 4, Several times a day; and PCA, QoL = 0, not at all; 1, a little bit; 2, some-what; 3, quite a bit; 4, very much (see Table 2).

**Table 2:** Functional Assessment of Cancer Therapy–Cognitive Function (FACT-Cog, Version 3)

Subscale	FACT-Cog (version 3)		
	Item ID	Item/question	Response options
PCI	CogA1	I have had trouble forming thoughts	Frequency (never, about once a week, two to three times a week, nearly every day, several times a day)
	CogA3	My thinking has been slow	
	CogC7	I have had trouble concentrating	
	CogM9	I have had trouble finding my way to a familiar place	
	CogM10	I have had trouble remembering where I put things, like my keys or my wallet	
	CogM12	I have had trouble remembering new information, like phone numbers or simple instructions	
	CogV13	I have had trouble recalling the name of an object while talking to someone	
	CogV15	I have had trouble finding the right word(s) to express myself	
	CogV16	I have used the wrong word when I referred to an object	
	CogV17b	I have had trouble saying what I mean in conversations with others	
	CogF19	I have walked into a room and forgotten what I meant to get or do there	
	CogF23	I have had to work really hard to pay attention or I would make a mistake	
	CogF24	I have forgotten names of people soon after being introduced	
	CogF25	My reactions in everyday situations have been slow	
	CogC31	I have had to work harder than usual to keep track of what I was doing	
	CogC32	My thinking has been slower than usual	
	CogC33a	I have had to work harder than usual to express myself clearly	
	CogC33c	I have had to use written lists more often than usual so I would not forget things	
OTH	CogO1	Other people have told me I seemed to have trouble remembering information	Frequency (never, about once a week, two to three times a week, nearly every day, several times a day)
	CogO2	Other people have told me I seemed to have trouble speaking clearly	
	CogO3	Other people have told me I seemed to have trouble thinking clearly	
	CogO4	Other people have told me I seemed confused	
PCA	CogPC1	I have been able to concentrate	Intensity (not at all, a little bit, somewhat quite a bit, very much)
	CogPV1	I have been able to bring to mind words that I wanted to use while talking to someone	
	CogPM1	I have been able to remember things, like where I left my keys or wallet	
	CogPM2	I have been able to remember to do things, like take medicine or buy something I needed	
	CogPF1	I am able to pay attention and keep track of what I am doing without extra effort	
	CogPCh1	My mind is as sharp as it has always been	
	CogPCh2	My memory is as good as it has always been	
Cog QoL	CogQ35	I have been upset about these problems	Intensity (not at all, a little bit, somewhat quite a bit, very much)
	CogQ37	These problems have interfered with my ability to work	
	CogQ38	These problems have interfered with my ability to do things I enjoy	
	CogQ41	These problems have interfered with the quality of my life	

Negatively worded items of the subscales (e.g., “I have had trouble forming thoughts”) were reversed in the calculation for the final score. The scoring key for all items is reversed except for the PCA subscale. Two items of PCA and PCI were not scored because these items, related to multitasking, have not yet been validated and incorporated with the current FACT-Cog scoring algorithm [18, 25].

### Statistical analysis/data analysis

The data analysis was performed with the statistical software package IBM SPSS statistics 26 (Chicago, IL, USA). Only those patients who completed all assessments were included in the analysis. Descriptive statistics are presented as mean, standard deviation (SD), and the minimum and maximum of the outcome parameters. A significance level of P < 0.05 for data analyses was set. Demographic characteristics (age, height, weight, BMI) were tested using ANOVA to ensure comparability between the study groups. All metric data were normally distributed (Shapiro–Wilk test) *p* > 0.05, and the null hypothesis was not rejected (shown in Table 3). For applying mixed (between-within) ANOVA, sphericity was identified (Mauchly test). Variances of the four study groups were equal (homogeneity) (Levene’s test). Univariate ANOVA with intermediate subject effects demonstrated no significant group differences on the dependent variables at T0 (*p* > 0.05). The main effects for time (whole group), the interaction between time and group (difference between groups), as well as group comparison regardless of the time, were tested for significant effects using a mixed ANOVA and posthoc analysis (Tukey, Games-Howell). Group differences over time were investigated with main effects of the between-subjects factor and secondary outcome variables with a repeated measure analysis of variance for main effects of the within-subject factor (Greenhouse–Geisser). F indicates that the test procedure uses an F statistic based on the F distribution.

**Table 3:** Baseline demographics and patients’ clinical characteristics of n = 79 women with breast cancer

Variable	Group SC	Group SCR	Group SR	Group S
N. (%)	22 (100.0)	17 (100.0)	27 (100.0)	13 (100.0)
Age [years]	51.9 ± 11.6	54.4 ± 8.5	56.7 ± 9.0	55.3 ± 7.3
*p*	0.30	0.21	0.06	0.28
Age, 30–35 years n (%)	2 (9.1)	0 (0.0)	0 (0.0)	0 (0.0)
Age, 35–40 years n (%)	2 (9.1)	0 (0.0)	3 (11.1)	0 (0.0)
Age, 41–49 years n (%)	5 (22.7)	6 (45.5)	2 (7.4)	3 (23.1)
Age, 50–59 years n (%)	6 (27.3)	4 (23.5)	9 (33.3)	6 (46.2)
Age, 60–69 years n (%)	7 (31.8)	7 (41.2)	13 (48.2)	4 (30.8)
Height [m]	1.65 ± 0.08	1.65 ± 0.08	1.61 ± 0.06	1.63 ± 0.08
*p*	0.43	0.77	0.56	0.24
Weight [kg]	72.1 ± 14.2	82.7 ± 20.2	68.6 ± 12.4	72.6 ± 12.5
*p*	0.29	0.11	0.66	0.55
BMI [kg m^−2^]	26.4 ± 5.0	30.5 ± 6.8	26.4 ± 4.8	27.4 ± 4.3
*p*	0.08	0.08	0.48	0.17
UICC n (%)	IA: 5 (22.7) IIA:10 (45.5) IIIA: 1 (4.6) IIB: 6 (27.3)	IA: 7 (41.2) IIA:7 (41.2) IB: 2 (11.8) IIB: 1 (5.9)	IA: 25 (92.6) IIA: 2 (7.4) IB: 0 (0.0) IIB: 0 (0.0)	IA: 5 (38.5) IIA: 8 (61.5) IB: 0 (0.0) IIB: 0 (0.0)
TT (month)	7.7 ± 1.3	10.4 ± 1.6	5.3 ± 1.5	2.5 ± 1.6
Her2/neu status, n (%)	Pos. 1 (4.5) Neg. 21 (94.5)	Pos. 2 (11.8) Neg. 15 (88.2)	Pos. 0 (0.0) Neg. 27 (100.0)	Pos. 13 (100.0) Neg. 0 (0.0)
ER status, n (%)	Pos. 13 (59.1) Neg. 9 (40.9)	Pos. 14 (82.4) Neg. 3 (17.7)	Pos. 27 (100.0) Neg. 0 (0.0)	Pos. 13 (100.0) Neg. 0 (0.0)
MC, n (%)	1 (4.6)	1 (5.9)	0 (0.0)	0 (0.0)
IDC, n (%)	18 (81.8)	16 (94.1)	24 (88.9)	7 (53.9)
IDC-L, n (%)	0 (0.0)	0 (0.0)	0 (0.0)	1 (7.7)
ILC, n (%)	3 (13.6)	0 (0.0)	2 (7.4)	5 (38.5)
ICC, n (%)	0 (0.0)	0 (0.0)	1 (3.7)	0 (0.0)
SNB, n (%)	21 (95.5)	15 (88.2)	27 (100.0)	13 (100.0)
ALND, n (%)	5 (22.7)	3 (17.7)	0 (0.0)	0 (0.0)
BCS, n (%)	8 (36.4)	16 (94.1)	27 (100.0)	0 (0.0)
MRM, n (%)	2 (9.1)	1 (5.9)	0 (0.0)	2 (15.4)
SCM, n (%)	9 (40.9)	0 (0.0)	0 (0.0)	10 (76.9)
BCS + SCM, n (%)	3 (13.6)	0 (0.0)	0 (0.0)	1 (7.7)
TMX, n (%)	1 (4.5)	4 (23.5)	6 (22.2)	1 (7.7)
ALs, n (%)	13 (59.1)	9 (52.9)	20 (74.1)	11 (84.6)
Neoadjuvant C, n (%)	10 (45.5)	9 (52.9)	0 (0.0)	0 (0.0)
Adjuvant C, n (%)	13 (59.1)	8 (47.1)	0 (0.0)	0 (0.0)
Anth-bCTx, n (%)	12 (54.6)	9 (52.9)	0 (0.0)	0 (0.0)
TaxAnth-C, n (%)	10 (45.5)	8 (47.1)	0 (0.0)	0 (0.0)
R, n (%)	0 (0.0)	17 (100.0)	27 (100.0)	0 (0.0)

Means ± standard deviation (SD); n = number of patients (%); normally distribution (Shapiro–Wilk test) *p* > 0.05; SCR, Surgery + Chemotherapy + Radiotherapy; SC; SR; S; ALND, Axillary lymph node dissection; ALs, Aromatase inhibitors; Anth-bC, Anthracycline-Based Chemotherapy; BCS, Breast-conserving surgery; ER, estrogen receptor; HER2/neu, human epidermal growth factor receptor 2; ICC, invasive cribriform carcinoma; IDC, Invasive ductal carcinoma; IDC-L, invasive ductal carcinoma with lobular features; ILC, invasive lobular carcinoma; MC, Mucinous breast carcinoma; MRM, Modified Radical Mastectomy; R, Radiotherapy; SCM, Subcutaneous mastectomy; SNB, Sentinel node biopsy; TMX, Tamoxifen; TaxAnth-C, Anthracycline-Taxane-Based Chemotherapy; TT, Time of Treatment; UICC, Union for International Cancer Control

Additionally, we performed the repeated-measures ANOVA for each group separately to inspect if the individual groups differed significantly (*p* < 0.05) between T0 and T1 (Greenhouse–Geisser). Significant interaction effects were reviewed with post hoc test Tukey-HSD for multiple comparisons, which allowed identification of groups differing from each at T0 and T1.

The effect size was calculated by using the formula: $partial {n}^{2}= \frac{{SS}_{effect}}{SSeffect + SSerror}$. Suggested benchmarks for interpretation of the effect size are small (0.1–0.3), medium (0.3–0.5) and large (> 0.5) [26]. Cronbachs alpha formula: $a= \frac{N\overline{c} }{ \overline{v }+\left(N-1\right) \overline{c} }$ provided reliability of used questionnaires on the sample in this research. The measured internal consistency for FACT B (0.76) and for FACT-COG (0.84) was good to very good [27].

## Results

The Federal Clinical Cancer Registry provided patients’ clinical characteristics through a comprehensive registration of tumor cases. Along with the assessed baseline demographics, data were displayed in Table 3.

Seventy-nine women (100%) with breast cancer were included in the present analysis. The mean (SD) age of the total sample at diagnosis was 54.6 ± 9.5 years (range = 30 to 69 years). The mean (SD) time interval between diagnosis of breast cancer and initial data collection before starting treatment for breast cancer (T0) was 6.8 ± 1.3 days (range 5.0—9.0 days). The written informed consent for participation in this study after diagnosis was given within 4.5 ± 1.2 days (range 2.0–7.0 days). All women with primary disease completed their cycles of chemotherapy, treatment sessions of radiation therapy, or cancer surgery. The mean (SD) time for completing therapy was 6.6 ± 3.0 months (range 1.0–13.4 months.). After breast cancer treatment, follow-up data were collected within one week (mean 5.7 ± 0.8 days, range 4.0 – 7.0 days). The length of treatment (TT) or treatment period was defined as the time from the day of diagnosis, including treatment initiation and the disappearance of all signs of cancer in response to treatment certified by the individual’s treating health care professional. Completing breast treatment does not always mean that cancer has been cured. A treatment period lasting more than twelve months requires a review of the need for continued treatment.

### Health-related quality of life

The longitudinal comparison indicated a significant main (time) effect on the FACT-B and all subscales except EWB, with medium to large effect size in FACT-B and BCS presented in Table 4. Regarding group allocation (individual group), T0 to T1 differences were not significant for all four groups (*p* > 0.05) on SWB, EWB, and FWB. The highest on average score change for each patient (percentage) was detected in SCR (PWB − 30%, BCS − 20%, SWB − 15%, FWB − 20% FACT-B − 19%) compared to S (PWB − 12%, BCS − 7%, SWB − 9%, FWB − 5%, FACT-B − 7%). All groups showed a more than 10% reduction in PWB. More than a 10% reduction was detected in FWB in SC, SCR, and SR. Significant group-by-time interaction for FACT-B, FWB, BCS showed more deterioration experienced with SC and SCR than SR and S. The effect size for the primary outcome variable was small.

**Table 4:** The primary outcome measures of the FACT-B and the FACT-Cog in the treatment subgroups

Variable	G	Mean (SD)		n	Change (%)	IG T*	F–T	F–G	F–GxT	η^2^ (T)	η^2^ (GxT)
		T0	T1								
FACT-B (0–148)	SC	114.5 ± 13.9	98.8 ± 14.7	22	− 13.2 ± 11.9	a	82.62***	0.17^NS^	4.12**	0.52	0.14
	SCR	116.7 ± 12.1	94.8 ± 18.9	17	− 18.6 ± 13.8	a					
	SR	113.5 ± 16.9	103.8 ± 19.2	27	− 8.8 ± 8.6	a					
	S	111.4 ± 9.1	103.5 ± 13.4	13	− 7.3 ± 7.5	a					
PWB (0–28)	SC	24.7 ± 2.1	19.8 ± 4.9	22	− 19.5 ± 20.5	a	70.47***	1.35^NS^	2.26^NS^	0.48	0.08
	SCR	23.9 ± 2.9	16.8 ± 6.3	17	− 29.7 ± 23.4	a					
	SR	24.0 ± 3.8	20.0 ± 5.2	27	− 16.7 ± 17.0	a					
	S	24.1 ± 2.8	21.3 ± 4.1	13	− 11.5 ± 12.9	a					
SWB (0–28)	SC	23.0 ± 3.4	20.2 ± 3.4	22	− 11.5 ± 14.4	a	36.35***	0.86^NS^	2.60^NS^	0.33	0.09
	SCR	24.8 ± 3.0	21.0 ± 3.9	17	− 15.3 ± 10.8	a					
	SR	22.4 ± 3.9	21.3 ± 5.0	27	− 4.2 ± 20.1						
	S	21.8 ± 3.5	19.9 ± 5.0	13	− 9.1 ± 17.2						
EWB (0–24)	SC	15.2 ± 4.8	15.7 ± 3.9	22	+ 7.7 ± 29.9		0.47^NS^	0.09^NS^	0.16^NS^	0	0
	SCR	15.8 ± 3.1	15.7 ± 3.5	17	+ 0.7 ± 20.1						
	SR	15.1 ± 4.6	15.5 ± 4.9	27	+ 3.4 ± 22.2						
	S	15.9 ± 2.9	16.0 ± 2.5	13	+ 1.3 ± 8.2						
FWB (0–28)	SC	19.4 ± 6.5	15.7 ± 5.4	22	− 16.7 ± 15.5	a	51.36***	0.33^NS^	3.25*	0.41	0.12
	SCR	20.9 ± 5.2	16.1 ± 4.3	17	− 19.8 ± 20.5	a					
	SR	20.0 ± 4.7	17.7 ± 4.4	27	− 10.3 ± 11.9	a					
	S	18.8 ± 3.8	17.5 ± 3.3	13	− 5.4 ± 12.5						
BCS (0–40)	SC	32.0 ± 3.5	27.5 ± 4.8	22	− 14.1 ± 12.3	a	76.11***	1.03^NS^	3.98*	0.5	0.13
	SCR	31.4 ± 4.4	25.2 ± 6.3	17	− 19.7 ± 14.8	a					
	SR	32.0 ± 4.1	29.3 ± 5.5	27	− 9.1 ± 10.7	a					
	S	30.8 ± 4.1	28.7 ± 5.0	13	− 6.8 ± 10.6	a					
FACT-	SC	118.1 ± 13.4	100.4 ± 11.8	22	− 14.9 ± 6.0	a	168.53***	1.64^NS^	27.93***	0.69	0.53
Cog (0–132)	SCR	117.9 ± 11.8	93.6 ± 13.8	17	− 20.5 ± 9.4	a					
	SR	116.3 ± 14.5	110.9 ± 18.4	27	− 5.1 ± 6.6	a					
	S	116.9 ± 18.6	114.5 ± 14.9	13	− 1.3 ± 6.5						
PCI (0–72)	SC	64.2 ± 10.0	56.7 ± 9.1	22	− 11.6 ± 5.0	a	118.70***	1.09^NS^	15.07***	0.61	0.38
	SCR	64.8 ± 7.4	52.9 ± 9.2	17	− 18.1 ± 11.6	a					
	SR	64.5 ± 7.7	61.1 ± 9.8	27	− 5.6 ± 7.1	a					
	S	64.5 ± 10.0	62.8 ± 8.3	13	− 2.2 ± 7.1						
OTH (0–16)	SC	15.5 ± 1.0	13.7 ± 2.0	22	− 11.9 ± 11.7	a	55.83***	1.14^NS^	10.82***	0.43	0.3
	SCR	15.4 ± 1.5	13.1 ± 1.7	17	− 15.0 ± 9.5	a					
	SR	15.6 ± 0.8	15.2 ± 0.9	27	− 2.0 ± 5.7						
	S	15.7 ± 0.6	15.4 ± 1.0	13	− 1.9 ± 5.1						
PCA (0–28)	SC	25.1 ± 3.2	20.6 ± 3.2	22	− 17.2 ± 11.4	a	91.45***	0.76^NS^	17.40***	0.55	0.41
	SCR	24.1 ± 3.2	19.6 ± 3.0	17	− 18.4 ± 7.8	a					
	SR	23.4 ± 4.8	22.6 ± 5.1	27	− 3.6 ± 8.3	a					
	S	24.2 ± 4.5	23.7 ± 4.0	13	− 1.7 ± 6.7						
QoL (0–16)	SC	13.3 ± 2.8	9.3 ± 1.9	22	− 28.2 ± 14.0	a	85.35***	1.44^NS^	22.49***	0.53	0.47
	SCR	13.6 ± 2.6	8.0 ± 1.9	17	− 39.2 ± 17.1	a					
	SR	12.8 ± 3.4	11.9 ± 4.2	27	− 8.2 ± 20.1						
	S	12.5 ± 4.6	12.7 ± 3.0	13	+ 1.8 ± 14.6						

Data are expressed as means ± standard deviation (SD); Change in percent (%) represent the average score change of each patient

For FACT-B and FACT-Cog, higher scores indicate a subjectively better HRQoL or PCF

SC, Surgery + Chemotherapy; SCR, Surgery + Chemotherapy + Radiation Therapy; SR, Surgery + Radiation Therapy; S, Surgery; n, number of patients; NS, not significant; T, time; G, group; F–T, main effect for time group; F–G, main effect for group regardless of the time; GxT, interaction between time and group; IG T, individual group for time

*p < 0.05; **p < 0.01; ***p < 0.001

"a" expresses statistically significant effects (*p* < 0.05) from basline to T1 of each group of the variable

All groups’ mean EWB (15.5 ± 3.9) represented around 65% of the total item score (maximum achievable amount of points; 24 points) at T0. FACT-B mean score of the overall group at T0 (114.0 ± 13.0) reached 73% of the maximum score (148 points) and 64% at T1 (FACT-B 100.2 ± 16.6). The overall group means for EWB and FACT-B do not report the average score change for each patient in time.

### Perceived cognitive function

Significant main effect (time) was shown in FACT-Cog; PCI; OTH; PCA; QoL, with large effects sizes in FACT-Cog and PCI. Individual groups (S, SR), T0 to T1 differences were not significant for all subscales (*p* > 0.05). Moreover, there was no significant effect detected in the time of S. The highest on average score change for each patient (percentage) was established for SCR (PCI − 18%, OTH − 15%, PCA − 18%, QoL − 39% FACT-Cog − 21%) compared to S (PCI − 2%, OTH − 2%, PCA − 2%, QoL + 2% FACT-Cog − 1%).

A significant group × time interaction for FACT-Cog; PCI; OTH; PCA; QoL was detected as SC and SCR presented more reductions than SR, and S. Testing partial n^2^ showed a medium to large effect size for FACT-Cog, PCI, OTH, PCA, and QoL. The results of the primary outcome measure, the FACT-B, and the FACT-Cog, are presented in Table 4.

FACT-Cog mean score of the entire group (117.3 ± 14.6) at T0 reached 89% of maximum score (maximum achievable amount of points; 132) and 80% at T1 (FACT-Cog 104.9 ± 14.7).

## Discussion

Based on the preliminary data of the research study “Return”, we analyzed the PRO measures HRQoL and PCF in women with breast cancer regarding their cancer treatments. Monitoring with the help of clinically established assessment procedures was conducted. Our main findings provide evidence of decreased HRQoL and PCF across all groups, with the most pronounced impact in SCR following multi-modular treatment. Differences were shown in the reduced FWB, BCS, PCI, PCA, OTH, and Cog QoL of women receiving additional chemotherapy.

The significantly reduced HRQoL and PCF of women in the presented study might reflect an increased demand for supportive care to compensate for the side effects of breast cancer treatment and throughout the different stages of therapy. The oncological care pathway in Germany includes acute medical treatment and voluntary follow-up rehabilitation in the clinical setting of three weeks immediately or at the latest three weeks after discharge of hospital treatment [28, 29].

Early post-diagnostic regular exercise with moderate-to-vigorous intensity may prevent unfavorable impairment of patients’ everyday lives and enhance tolerance to medical cancer treatments. As a complementary measure, it could contribute to satisfying therapy goals on physical function (e.g., increasing stamina, reducing restrictions). Further successful reintegration into working life and avoiding certain lifestyle factors, e.g., diet, smoking, alcohol consumption, might be facilitated. Exercise sessions carried out by specialist therapists show substantial advantages over unsupervised physical activities [30–33].

A reduction of 2–3 points in the FACT-B subscales is considered a meaningful change that patients perceive as harmful, leading the clinician to initiate modifications [24, 34]. Care practitioners with different specialties such as a nurse, medical doctor, pathologist, oncologist, radiotherapist, psycho-oncologist, physician assistant, pharmacist, and physical therapist should decide in regular multidisciplinary tumor conferences (MTCs) on cancer patient’s management plans. The exchange of best practices for this type of care is multifactorial and requires extra effort in coordination, communication, and cooperation between health care providers [35].

Systematic observing PRO with the FACT-B was feasible in patients with different treatment conditions. Moreover, due to its reliability and time efficiency, identifying patients with decreased quality of life scores within one week after completing cancer therapy turned out to be possible. Thus patients were continuing endocrine therapy persisting adverse events may be expected, which warrants closer examination beyond the time frame chosen in this study to prevent further decline [36]. In this context, late-occurring manifestations associated with radiotherapy, e.g., coronary artery disease, pericarditis, and myocardial dysfunction, have to be considered [37, 38].

Although there is rising awareness of capturing treatment-related QoL of breast cancer patients in scientific studies, monitoring PROs are not yet part of routine oncology practice [15, 16]. This leads to the assumption that disorders are not consistently recognized. Dismissing patient experiences as an understandable reaction to a life-threatening illness may reduce the success of therapeutic outcomes, which in the worst case negatively affects breast cancer prognosis [39]. Patients who underwent adjuvant chemotherapy experienced significantly unmet sexuality needs, poor physical, functional well-being, and more severe breast cancer-specific concerns. It transpired that details of treatment modalities lead to a more diversified assessment of the patient’s perceived situation.

Based on the response behavior, dimensional reductions were detected. A lower PWB at T1 was associated with pain, lack of energy, illness, and being forced to spend time in bed. Some of the poor PWB and BCS incidences may be short-term and were related to all treatments, particularly surgery and radiation therapy. Nonetheless, a significant decrease in BCS is accompanied by an inability to feel like a woman. These results may be related to possible chemotherapy-induced menopausal symptoms, influencing sexual interest and desire [40, 41].

Furthermore, the long time of receiving cancer treatment and younger age may increase lifestyle stresses, such as lack of ability to work, child care, or elderly care. Additionally, women felt anxious by changing weight, hair loss, and swollen arms. These findings are attributed to the cancer-specific drug [42] combined with surgical treatment [43]. Studies showed that about 40% of women with lymph node removal followed by radiation therapy develop secondary lymphedema. Participation in regular physical activity as soon as possible is recommended to stimulate lymphatic circulation, preferably 2–3 weeks after surgery [44]

In consequence of significantly reduced FWB, patients had a hard time accepting the illness or could not work and enjoy daily activities (e.g., I am enjoying the things I usually do for fun). It is very likely that a fatigue disorder occurred, described by multifactorial symptoms [45]. Supportive forms such as pain therapy [46], nutritional medicine [47], exercise therapy (e.g., yoga)[48] should be implemented in breast cancer patients after completion or during cancer treatment. Especially women receiving chemotherapy reported reduced satisfaction with their sex life, communication about the illness, and experiences of less support from family and friends (SWB). Avoiding discussing the challenges of cancer may become burdensome [49]. Most patients prefer to receive information from a nurse or primary care provider about the impact on intimacy and sexuality shortly after treatment starts [50]. Breast cancer patients receiving active treatment can face many social-emotional challenges and limiting consequences in the HRQoL related to surgical treatment. The increased fear of recurrence, cancer-related distress and body image dissatisfaction are reflected in significantly lower scores.

A low EWB at T0 and T1 was linked to all study groups and may indicate the presence of mental comorbidities. A more substantial alignment with worries about dying and the deterioration of conditions appears necessary, while the attitude of helplessness and hopelessness is progressing. Transparency on mental disorders and identifying patients at risk is all too often lacking [51, 52].

We found reduced PCF prior to cancer treatment in the subgroups using the FACT-Cog to evaluate changes. Differences may be explained by emotional distress associated with the breast cancer diagnosis, causing disrupted functional dynamics [53, 54].

Patients receiving chemotherapy experienced an adverse effect on verbal fluency and processing information. Additionally, a majority of people with whom they were interacting told them that they might have trouble thinking clearly or seemed to be confused. The restrictive PCI impacted QoL (e.g., “I have been upset about these problems”) and may fuel a vicious cycle of tighter capital, job losses, and the inability to handle instrumental activities of daily living. Concerns about completing education or meeting job requirements to secure financial stability can be debilitating, leading to withdrawal from social life to avoid stigmatization [55]. Women need to receive information about the possible effects of memory loss and advice about coping methods [56].

Underlying mechanisms resulting in lower PCF might be influenced by the state of an inflammatory tumor [57], changes in hormone levels [58], attentional fatigue, and neurotransmitter deregulation [59]. Findings within the chemotherapy-exposed groups may be traced back to neurotoxicity causing neurologic damage [60], cancer-related cognitive impairment (CRCI) [12, 61], and interhemispheric transfer deficits [62]. However, the origin of CRCI often remains unclear and is not fully understood.

Exercise therapy interventions, individually adapted to the fitness and treatment phase, may counteract the loss of self-confidence and help cope with illness-related symptoms [63]. Frequently exercising of overweight breast cancer survivors showed a positive relationship between BMI and PCI [64].

Nonetheless, the evidence of studies focusing on improving CRCI with exercise treatment is limited [65, 66]. In the light of apparent cognitive deficits, study designs should employ baseline assessments to detect changes accurately. More research is needed to identify how clinical characteristics, including older age, obesity, dietary supplements, stage of cancer, and side effects of chemotherapy, contribute to an increased risk of impairment.

This study adds to the existing literature on patient experiences of cancer care. A clear benefit was that patient-orientated indicators could be assessed quickly and efficiently. By carefully comprehending patients’ treatment conditions, prospective capturing of patient-perceived circumstances may be improved. The present study results show the importance and the need for differencing between treatments. Studies in larger populations are necessary to guide support based on the medical intervention received.

Knowledge of treatment-specific PRO could serve as a basis for decision-making. The challenge, of course, is to shift from an intuitive approach based on the individual situation to an evidence-based one described by a large number of patients with similar clinical characteristics. This means that every woman with breast cancer could benefit from the generalizable findings of a larger cohort.

Assessing the PCF with the FACT-Cog and the HRQoL with FACT-B should be integrated as a standard measure in women with breast cancer for advanced classifications and standardized definitions of CRCI and health status. Measuring HRQoL and PCF may give multidisciplinary health care professionals guidance for determining the individualized needs of women with breast cancer. The most appropriate supportive care modalities and timing for implementation are required for a beneficial approach in subsequent oncological rehabilitation treatment.

### Limitations

There are limitations to this study as we could not include an additional follow-up analysis. The findings can only be considered preliminary since the number of patients, especially in S, was small (n = 13). Future investigations are necessary for the generalizability of our results. Treatment groups may not represent all cancer patients, especially not those with severe course of illness. Socioeconomic status, age, BMI, and UICC stage were not investigated as possible modifying factors. A multivariable risk-stratified approach identifying causal inference of reduced FACT-B and FACT-Cog may upgrade the interpretation of data. Measurements in sexuality represent unmet needs and discontent; hence this study’s results cannot be compared with those of other studies regarding sexual function.

## Conclusion

In summary, women with breast cancer presented a decreased HRQoL and PCF across all groups, with the most pronounced deterioration in SCR following a multi-modal treatment. Significant group-by-time interaction was particularly noticeable in FWB, BCS, PCI, PCA, and Cog QoL. Based on our findings, multidisciplinary support initiated early in breast cancer therapy, especially for women undergoing combined cancer treatment, is needed. Permanent adoption of PRO in oncology practice may increase the transparency of patients’ perceived circumstances, leading to personalized and optimized acute and survivorship care.

## Data Availability

The datasets used or/and analyzed during the current study are available from the corresponding author on reasonable request.
